# A ‘selfish’ B chromosome induces genome elimination by disrupting the histone code in the jewel wasp *Nasonia vitripennis*

**DOI:** 10.1038/srep42551

**Published:** 2017-02-13

**Authors:** John C. Aldrich, Alexandra Leibholz, Manjinder S. Cheema, Juan Ausiό, Patrick M. Ferree

**Affiliations:** 1 W. M. Keck Science Department, Claremont McKenna, Pitzer, and Scripps Colleges, Claremont, CA 91711, USA; 2 Department of Biochemistry and Microbiology, University of Victoria, Victoria, BC V8W-3P6, Canada

## Abstract

Intragenomic conflict describes a phenomenon in which genetic elements act ‘selfishly’ to gain a transmission advantage at the expense of the whole genome. A non-essential, selfish B chromosome known as Paternal Sex Ratio (PSR) induces complete elimination of the sperm-derived hereditary material in the jewel wasp *Nasonia vitripennis*. PSR prevents the paternal chromatin from forming chromosomes during the first embryonic mitosis, leading to its loss. Although paternally transmitted, PSR evades self-elimination in order to be inherited. We examined important post-translational modifications to the DNA packaging histones on the normal genome and the PSR chromosome in the fertilized embryo. Three histone marks – H3K9me2,3, H3K27me1, and H4K20me1 – became abnormally enriched and spread to ectopic positions on the sperm’s chromatin before entry into mitosis. In contrast, other histone marks and DNA methylation were not affected by PSR, suggesting that its effect on the paternal genome is specific to a subset of histone marks. Contrary to the paternally derived genome, the PSR chromosome was visibly devoid of the H3K27me1 and H4K20me1 marks. These findings strongly suggest that PSR causes paternal genome elimination by disrupting at least three histone marks following fertilization, while PSR avoids self-elimination by evading two of these marks.

In certain cases, individual elements within the cytoplasm or nuclear genome can act ‘selfishly’ to gain higher-than-Mendelian transmission at the cost of the genome as a whole. Causal elements of this condition, referred to as intragenomic conflict, range from single loci to whole chromosomes, and their ‘drive’ phenotypes often involve disruption of nuclear processes involved in gametogenesis or early embryogenesis^1^,^2^,^3^,^4^.

One of the most striking examples of intragenomic conflict stems from a non-essential, supernumerary B chromosome known as PSR (for Paternal Sex Ratio), which is present in natural populations of the jewel wasp *Nasonia vitripennis*^5^,^6^. PSR is transmitted to new progeny as a component of the sperm’s nuclear material. In PSR’s presence, the sperm-derived chromatin fails to form into individualized chromosomes during the first mitotic division of the embryo and is excluded from subsequent divisions^5^. In contrast, PSR itself escapes the paternal chromatin mass (PCM) during anaphase of the first division and segregates successfully with the unaffected egg-derived chromosomes^7^. In diploid organisms, elimination of half the genome would result in early embryonic lethality. However, *N. vitripennis* and all other hymenopteran insects (*i.e*., wasps, bees and ants) reproduce through haplodiploidy, in which males develop as haploids from unfertilized eggs while females arise as diploids from fertilized eggs^8^. Thus, PSR-induced elimination of the sperm-derived genome compliment converts female-destined embryos into males that carry and transmit PSR. Broadly, this effect leads to severely male-biased wasp populations, an effect that inspired the naming of PSR when it was originally detected^5^,^9^.

Two outstanding and interrelated aspects of this phenomenon are how the presence of PSR leads to blockage of normal chromosome resolution during the first mitosis, and how PSR excludes itself from elimination. More generally, how can a single chromosome target the rest of the genome for elimination while not targeting itself, when indeed both entities consist of chromatin and are components of the same nucleus?

An important part of addressing this question is to more fully understand the chromatin state of the paternal genome before and during its elimination. A previous study found that following fertilization the PSR-carrying paternal nucleus migrates normally to a position proximal to the maternal nucleus, and both sets pass successfully through a single round of DNA replication (S-phase) and enter together into the first mitosis^7^. However, the paternal chromatin fails to lose its phosphorylation of histone H3 at Serine residue 10 (H3S10p) at the end of this first division^7^. This mark is believed to be necessary for proper condensation and resolution of chromatin into chromosomes at the onset of nuclear division through the activity of Condensin^10^,^11^,^12^. Interestingly, abnormal retention of Condensin accompanies this H3S10p pattern^7^. Thus, failure of chromosome resolution in this condition could result specifically from inappropriate behavior of Condensin, which in turn may stem from retention of H3S10p. What remains to be determined, however, is whether PSR directly causes these chromatin defects in order to block chromosome resolution of the paternal set or, instead, acts indirectly through disruption of other upstream chromatin-based processes.

In this study we sought to address this question by visualizing a set of major chromatin state changes that occur specifically on the paternal set immediately following fertilization in PSR-carrying embryos. Normally, in most animals the sperm’s DNA is stripped of male-specific non-histone proteins known as protamines immediately after fertilization and concomitantly repackaged with conventional histones^13^,^14^,^15^. Through a process that is not well understood, certain post-translational histone marks, including, but not exclusive to, acetylation, methylation, and phosphorylation, appear on the paternal set in order to define different chromatin sub-types^16^. For example, acetylation of different Lysine residues in the tails of histones H3 and H4 comprise one of the first mark types to appear on the paternal chromatin as it is being remodeled with histones; acetylation of certain Lysine residues is retained and may help to structurally define regions of chromatin that are primed for active transcription at later developmental times^17^. In contrast, di- and tri-methylation of histone H3 at Lysine residue 9 (H3K9me2 and H3K9me3) appear at later mitotic cleavage divisions and help to establish a denser, transcriptionally quiescent type of chromatin known as constitutive (*i.e*., permanent) heterochromatin located in the pericentromeric regions^18^,^19^. These and other histone marks help to define chromatin sub-types through interactions with different non-histone structural proteins and histone-modifying enzymes^20^,^21^. We reasoned that perturbation of these or other important histone marks by PSR could in principle alter the chromatin state of the paternal set such that H3S10p and Condensin behavior are subsequently disrupted, thereby causing blockage of chromosome condensation and, thus, paternal genome elimination. We also speculated that any chromatin state change(s) that are crucial for paternal genome elimination would likely not apply to PSR because it resolves and segregates normally.

Our study identified three specific histone marks – H3K9me2,3, H3K27me1, and H4K20me1 – that become disrupted by PSR during paternal chromatin remodeling immediately after fertilization. In contrast, other marks, including acetylated histone H4, appeared normal during this developmental period. We also found that replication licensing factors appear on the paternal chromatin during the first S-phase, before the perturbed histone marks become visible, but not during the second S-phase, following these chromatin defects, thereby explaining why DNA replication fails specifically after the first mitotic division and not before. Interestingly, H3K27me1 and H4K20me1 were largely absent from the PSR chromosome, implicating these marks in particular as important for elimination of the paternal chromatin and evasion of this effect by PSR. Together these observations suggest that PSR disrupts the ‘histone code’ by altering a combination of histone marks in order to cause paternal genome elimination.

## Results

### PSR alters three post-translational histone modifications before entry into mitosis

**Figure 1:**
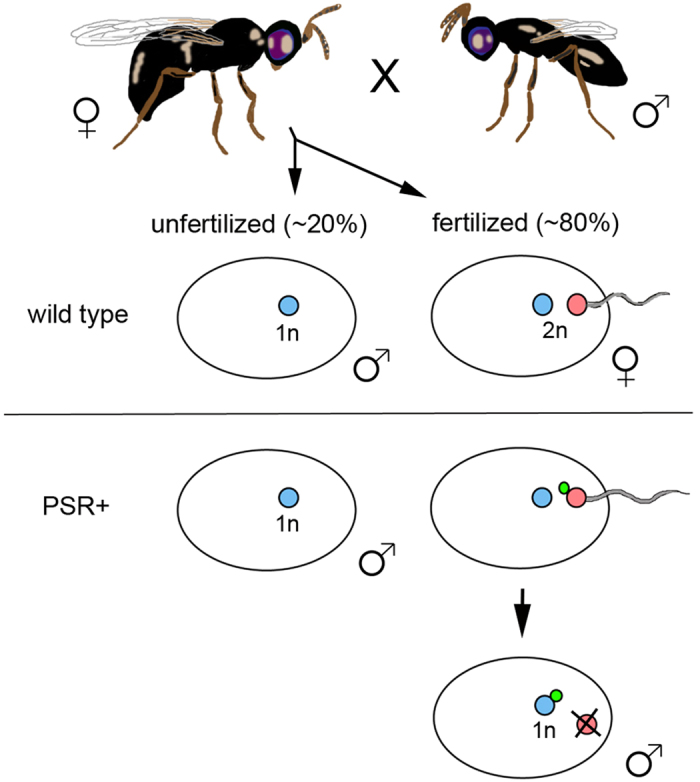
Haplodiploidy and its alteration by PSR. (Top) Mated *N. vitripennis* females naturally fertilize ~80% of eggs, which develop as diploid female embryos from genetic contribution of the sperm and egg nuclei (blue and red dots, respectively). The unfertilized eggs develop as haploid male embryos, with genetic contribution from only the egg. (Bottom) Females when mated with PSR-carrying males still fertilize ~80% of their eggs. However, the sperm-contributed genome, which harbors the paternally-transmitted PSR chromosome (green dot), fails to condense into chromosomes during the first mitosis and is lost during subsequent divisions. In contrast, the PSR chromosome associates with the egg-derived chromatin and segregates successfully. These events convert all fertilized eggs, which should become female, into PSR-transmitting male embryos.

In order to better understand the nature of paternal genome elimination by PSR, we used fluorescent-based tools to microscopically visualize the dynamics of several key post-translational histone modifications in young wild type and PSR-carrying embryos. In haplodiploid reproduction, females lay a combination of fertilized and unfertilized eggs, which normally develop into females and males, respectively^8^,^22^ (Fig. 1). Because PSR-induced genome elimination only occurs in fertilized embryos (Fig. 1), we excluded from our analyses unfertilized embryos. Instead, we focused entirely on fertilized embryos. Additionally, we compared the pattern of each chromatin mark on the PSR-carrying paternal set relative to patterns on (*i*) the maternal set in the same PSR-carrying embryos and (*ii*) the paternal set in wild type embryos, both of which should show normal patterns.

**Figure 2:**
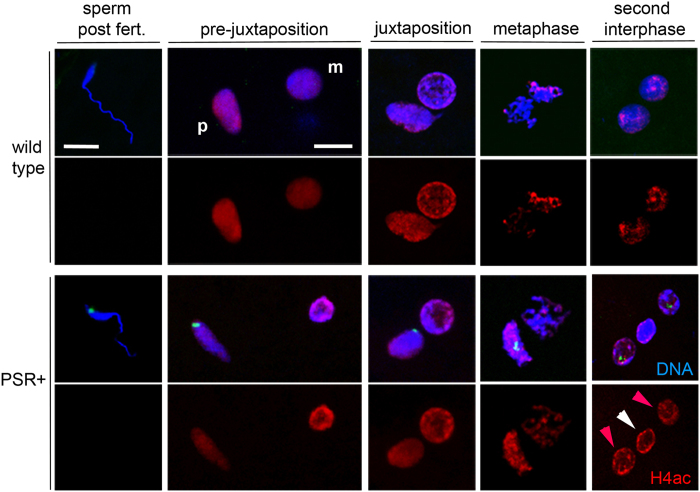
Acetylated histone H4 patterns are normal in PSR-carrying embryos. In wild type embryos, histone H4 acetylated at multiple Lysine residues is absent from the sperm’s chromatin immediately after fertilization. However, H4ac appears on the paternal chromatin as the sperm and egg nuclei migrate toward one another (pre-juxtaposition); the maternal nucleus and meiotic products (not shown) already show H4ac. This signal increases slightly more on the paternal set compared to the female set during metaphase, but by the end of the first mitosis both daughter nuclei contain similar levels of H4ac. In PSR-carrying embryos the patterns are indistinguishable. The paternal chromatin mass (PCM, indicated by white arrowhead in the bottom right panel) contains the same level of H4ac as the maternally derived nuclei (red arrowheads). PSR is highlighted by a sequence-specific FISH probe (green in panels of the bottom two rows). Scale bar equals 5 μM in the top left panel and 12 μM in the adjacent panel to the right, under pre-juxtaposition. p and m stand for paternal and maternal nuclei.

We began by examining the pattern of acetylated histone H4 because in *D. melanogaster* this mark is one of the first to appear on the paternal chromatin as it becomes remodeled with conventional histones^15^. In wild type embryos, H4ac was present on the egg’s meiotic products (not shown) but not on the highly elongated sperm nucleus (Fig. 2). This mark began to appear on the paternal chromatin as the two nuclei migrated toward one another; at this time the sperm’s nucleus widened into a more oval-like shape, also indicative of chromatin remodeling (Fig. 2)^16^. During juxtaposition, H4ac became equally intense on both nuclei (Fig. 2). During metaphase this mark appeared slightly brighter on the paternal set, which could be identified as faintly more condensed than the maternal set (Fig. 2). However, at the end of this first mitotic division, the two diploid daughter nuclei appeared indistinguishable in H4ac intensity (Fig. 2). In PSR-carrying embryos, the H4ac patterns were very similar to those in wild type. Additionally, the H4ac mark was visibly indistinguishable between the paternal chromatin mass and the maternally derived nuclei at the end of the first mitosis (Fig. 2). Thus, PSR does not visibly affect H4ac on the paternal half of the genome. Normal behavior of this mark and the passage of the paternal set through the first S-phase^7^ indirectly suggest that protamine removal occurs properly. Additionally, we conclude that, because the antibody used to detect H4ac recognizes multiple acetylated Lysine residues on histone H4, it is likely that each of these individual marks are largely unperturbed by PSR.

**Figure 3:**
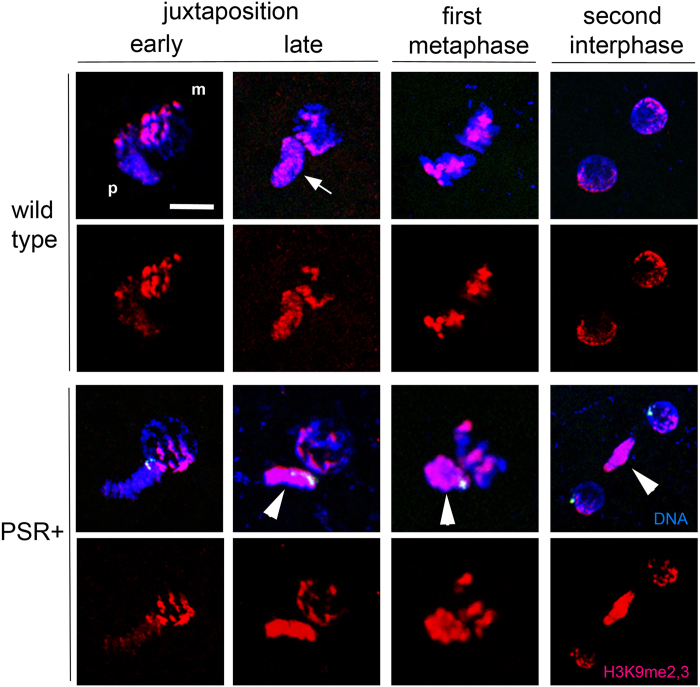
PSR disrupts histone H3K9me2 and H3K9me3 patterns on paternally-derived chromatin. In wild type embryos H3K9me2,3 begins to appear in localized regions of the paternal chromatin following juxtaposition. The signal persists in these regions while increasing in intensity (white arrow) until they are similar to regions on the maternal chromatin. This mark persists during metaphase, and obtains a RABL-like confirmation in the pericentromeric regions on opposing sides of the two daughter nuclei at the end of the first mitosis. In PSR-carrying embryos, the paternal chromatin obtains abnormally high levels of H3K9me2,3 across its entirety instead of in distinct regions (white arrowheads). This pattern persists following the end of the first mitosis. Scale bar is 10 μM.

We next examined H3K9me2 and H3K9me3 with special interest because they are hallmarks of constitutive heterochromatin^19^, and it has been previously suggested that formation of the PCM could involve heterochromatinization^7^. In this experiment we employed an antibody that recognized both marks, which we refer to collectively as H3K9me2,3. In wild type embryos, H3K9me2,3 was not observed on needle-shaped sperm nuclei immediately following fertilization (not shown) but began to appear in very small, patchy foci when the paternal and maternal nuclei had juxtaposed (Fig. 3). These foci became larger and more intense through metaphase, but they remained restricted to the pericentromeric regions (Fig. 3). This pattern is in contrast to those in *D. melanogaster*, in which H3K9me2,3 appears during later cleavage divisions^18^. At the end of mitosis H3K9me2,3 was present only in regions at the outer edges of the daughter nuclei, reminiscent of chromocenters, or coalesced pericentromeric regions. Additionally, this pattern of H3K9me2,3 is reflective of a RABL-like confirmation, in which the pericentromeric regions cluster together at the outer edges of two daughter nuclei at the end of nuclear division^23^. In PSR-carrying embryos, H3K9me2,3 began to appear during nuclear juxtaposition like in wild type embryos (Fig. 3). However, these marks became highly abnormal subsequently. Specifically, H3K9me2,3 spread across the entire paternal chromatin before entry into mitosis (Fig. 3). Additionally, H3K9me2,3 persisted strongly across the paternal chromatin even after exit from mitosis, when the two maternally derived haploid nuclei showed H3K9me2,3 only in their pericentromeric regions (Fig. 3). We also confirmed these abnormal patterns with antibodies that individually recognize H3K9me2 and H3K9me3 (Supplemental Figure 1).

In addition to H4ac and H3K9me2,3, we visualized four other histone marks for which we were able to obtain reagents suitable for immunofluorescence microscopy. Two of these marks, H3K27me1 and H4K20me1, were perturbed by PSR in a manner that closely mirrored H3K9me2,3 (Supplemental Figures 2 and 3). Two other marks, H3K27me2,3 and H3K4ac, appeared unperturbed by PSR; specifically, H3K27me2,3 was visible only on the maternal chromatin but not on the paternal chromatin in both wild type and PSR-carrying embryos, whereas H3K4ac, like H4ac, was present at low but comparable levels on both the PCM and maternally derived chromosomes (Supplemental Figure 1). Based on these results, we conclude that PSR strongly and broadly affects the chromatin state through alteration of multiple different histone marks immediately following the onset of paternal chromatin remodeling. However, because H3K27me2,3 and different histone acetylation marks are not visibly perturbed, the chromatin alteration is somewhat specific. Additionally, because marks other than H3K27me2,3 are perturbed, PSR may only affect histone marks that are normally placed onto the paternal chromatin following its repackaging after fertilization.

### Paternal genome elimination does not involve ectopic heterochromatinization or gross alteration of DNA methylation

**Figure 4:**
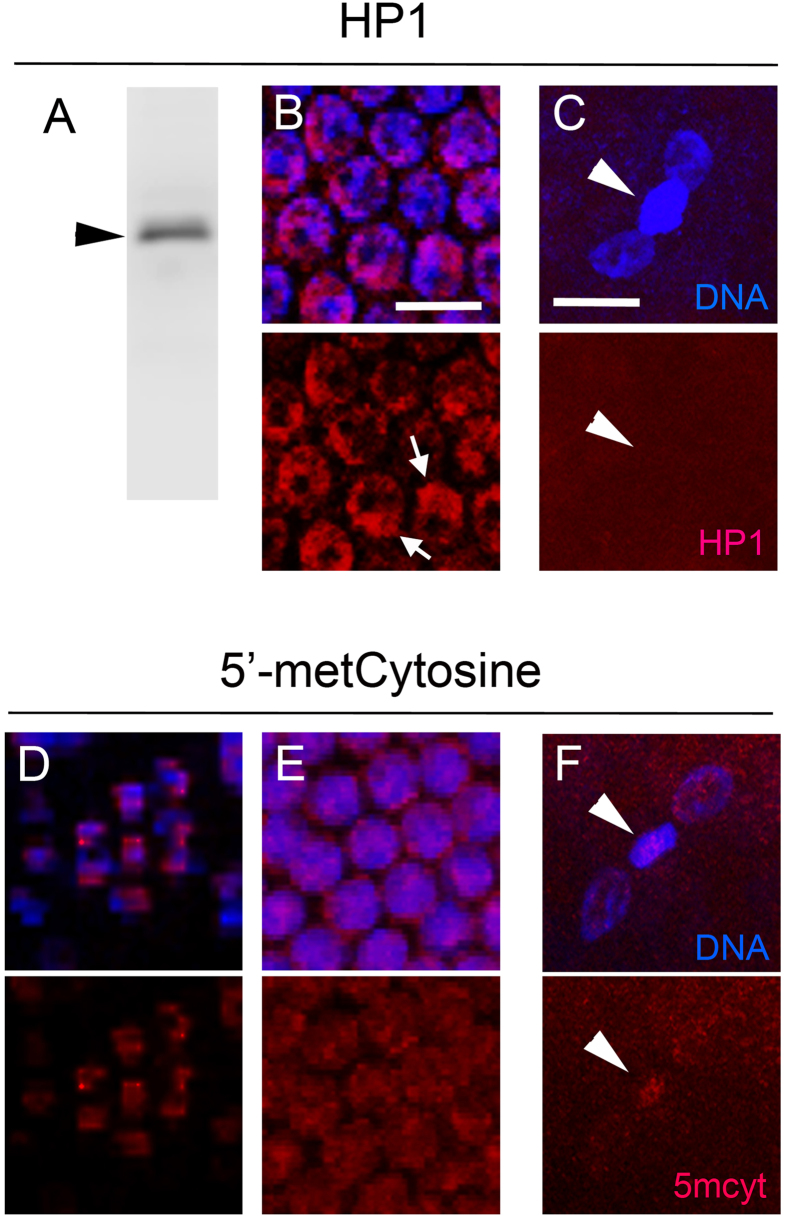
PSR does not cause ectopic heterochromatinization of the paternal chromatin or grossly affect its 5′-methylCytosine pattern. (**A**) A polyclonal serum raised against the single *N. vitripennis* HP1 protein recognizes a single band of 24 kDa (black arrowhead), its predicted mobility size, by Western blot. (**B**) The serum highlights bright, chromocenter-like regions (white arrows) within the nuclei of embryos in late cleavage. (**C**) However, no HP1 is present on either the PCM (white arrowheads) or maternally derived daughter nuclei following the first mitotic division. A monoclonal antibody highlights low but visibly detectable levels of 5′-methylCytosine in the nuclei of spermatogonia (**D**) and late cleavage embryos (**E**). (**F**) 5′-methylCytosine levels were slightly enriched on the PCM (white arrowhead) compared to the maternally derived nuclei at the end of the first mitotic division. Scale bars in (**B**) and (**C**) equal 10 μM and 12 μM, respectively.

We entertained the possibility that PSR causes abnormal heterochromatinization of the paternal chromatin in order to induce failure of chromosome resolution, a hypothesis that is consistent with abnormal spreading and persistence of H3K9me2,3 across the paternal chromatin. In order to test this possibility, we produced an antibody against the single *N. vitripennis* heterochromatin protein 1 (HP1) for cytological analysis^24^ (see Materials and Methods). Across the eukaryotes, the paralog HP1A associates with H3K9me2,3, a highly conserved interaction believed to contribute to the compactness and transcriptional silencing of heterochromatin^10^,^25^,^26^. Our antibody recognized a single band of approximately 24 kDa, the predicted size of the *N. vitripennis* HP1 protein, by Western blot (Fig. 4). Additionally, in late stage embryos this reagent showed a nuclear signal, with regions of strong localization that resembled chromocenters (Fig. 4). Thus, cytologically the *N. vitripennis* HP1 behaves similarly to HP1A in *D. melanogaster*. In young embryos HP1 did not localize to either the PCM or the maternally derived nuclei during or immediately following the first mitotic division (Fig. 4). This result is consistent with previous findings in *D. melanogaster* that HP1 does not begin to localize to chromatin until the late embryonic cleavage divisions^27^. Therefore, we conclude that elimination of the paternal chromatin does not involve heterochromatinization through ectopic localization of HP1.

We also visually examined 5′-cytosine methylation (5meCyt) on paternal chromatin. This chemical modification to DNA is commonly associated with governance of gene regulatory regions epigenetically and sometimes in a parent-of-origin manner^28^,^29^,^30^. Interestingly, 5meCyt has been functionally linked with H3K9me3^31^. Moreover, in the fungal gnat *Sciara coprophila*, high levels of 5meCyt were previously observed on the paternally inherited X chromosome before its programmed elimination during the cleavage divisions^32^. To visualize 5meCyt patterns in PSR-carrying wasp embryos we used an antibody that specifically recognizes this mark. For controls we also stained testes and late stage embryos from wild type animals because numerous genes are known to be methylated in *N. vitripennis* adult tissues^33^,^34^. Using conditions that denature double-stranded DNA, we were able to visibly detect 5meCyt signal that appeared to be heterogeneous within the nuclei of spermatogonia, the precursors of sperm, in testes of both genotypes (Fig. 4). We saw a similar heterogeneous signal in the nuclei of PSR-carrying embryos during late cleavage divisions (Fig. 4). Embryos at the end of the first mitotic division showed slightly elevated 5meCyt signal on the PCM compared to barely visible levels present on the maternally-derived haploid nuclei (n = 8/10 embryos) (Fig. 4). However, this increase in 5meCyt on the PCM is not nearly as pronounced as the abnormal histone marks, and may reflect the 2N ploidy of the PCM compared to the 1N ploidy of the maternally derived nuclei instead of an increase caused by PSR. Moreover, sperm nuclei from wild type and PSR-carrying males showed similar frequency and intensity of 5meCyt staining immediately following fertilization (n = 12/14 nuclei from wild type males; 9/11 nuclei from PSR-carrying males). Thus, we conclude that PSR does not substantially, if at all, disrupt 5 meCyt patterns on the paternal genome during sperm formation or during the first mitotic division.

### PSR-induced chromatin defects alter replication licensing of the second S-phase

It was previously shown that PCNA, a marker of active DNA replication, is distributed normally in the presence of PSR, suggesting that the paternal chromatin successfully undergoes S-phase preceding the first mitotic division^7^. However, the paternal chromatin does not become PCNA positive during the second S-phase, suggesting a failure of active replication during this second round^7^. To better understand these observations, we visualized different replication licensing factors on the PCM. One antibody used for this purpose was directed against ORC2, which together with five other ORCs (ORC 1 and ORCs 3-6) are responsible for initial recognition of the origins of replication. We also employed another antibody that recognizes the MCM factors 2 through 7 (MCM2-7); these proteins localize to the origins following ORC binding in order to complete the licensing phase so that replication can proceed^35^.

**Figure 5:**
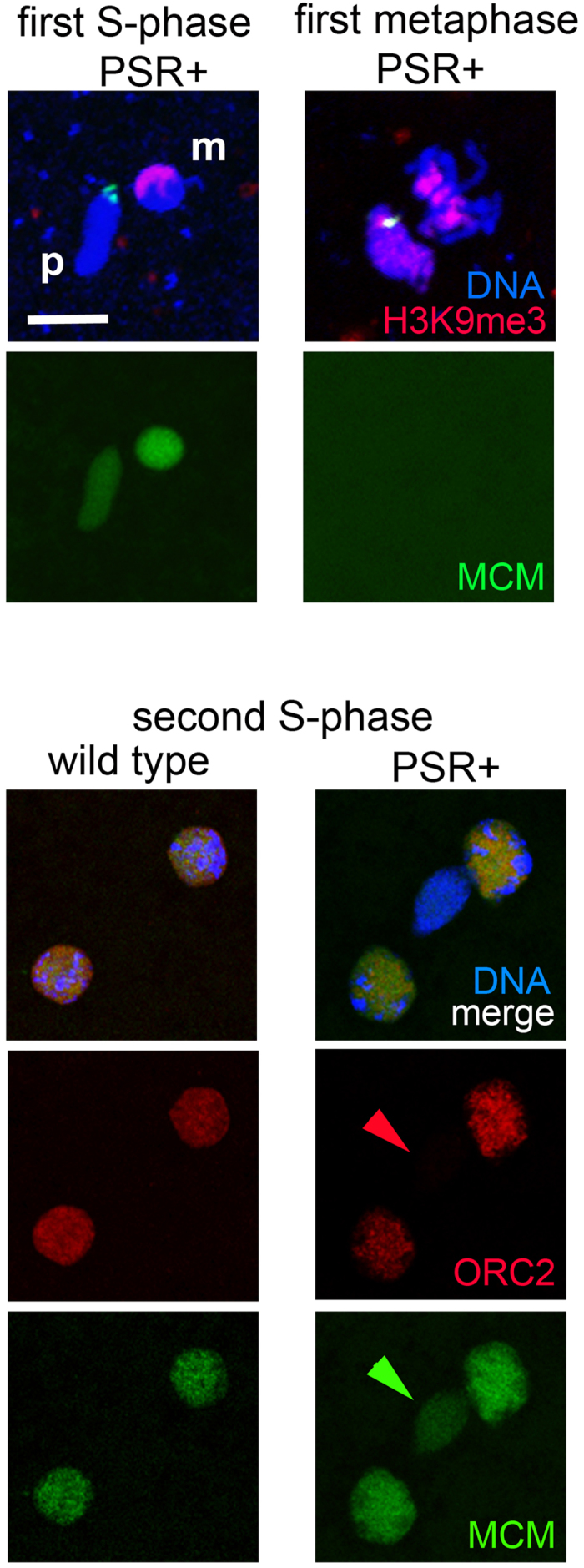
In the presence of PSR replication licensing factors localize properly to the paternal chromatin during the first but not the second S-phase. Top, PSR-carrying embryos during the first S-phase (left column) and metaphase of the first mitosis (right column) showing localization patterns of replication licensing factors, MCMs 2-7. Embryos were counterstained for H3K9me2,3 to show the initial appearance of this mark, which occurs after replication licensing. Bottom, wild type (left column) and PSR-carrying (right column) embryos stained for MCM2-7 and ORC2. While both factors localize properly to nuclei in wild type embryos and in maternally derived nuclei in PSR-carrying embryos, ORC2 was visibly absent from the PCM (red arrow) and MCMs were reduced on the PCM. Scale bar equals 12 μM. (p) and (m) mark the paternal and maternal nuclei, respectively.

We first confirmed normal progression of the first S-phase by co-staining young PSR-carrying embryos for MCM2-7 and H3K9me2,3. Consistent with previous PCNA patterns^7^, the PSR-carrying paternal chromatin obtained MCMs similarly to the maternal chromatin and before H3K9me2,3 became visible (Fig. 5). This pattern suggests that the first S-phase occurs normally because it happens immediately before the aberrant histone mark patterns appear. During metaphase, no MCMs were present on either paternal or maternal sets (Fig. 5), consistent with proper behavior of these factors (refs). In PSR-carrying embryos, both replication factors appeared on the maternally derived haploid nuclei during the second S-phase, similarly to diploid nuclei of wild type embryos (Fig. 5). However, the PCM was completely devoid of ORC2 and showed visibly reduced levels of MCMs (Fig. 5). This finding strongly suggests that failure of the paternal chromatin to pass through the second S-phase is due to improper replication licensing at this specific round of replication, an effect that may occur due to the PCM’s defective chromatin state at that time.

### The PSR chromosome is devoid of H3K27me1 and H4K20me1

**Figure 6:**
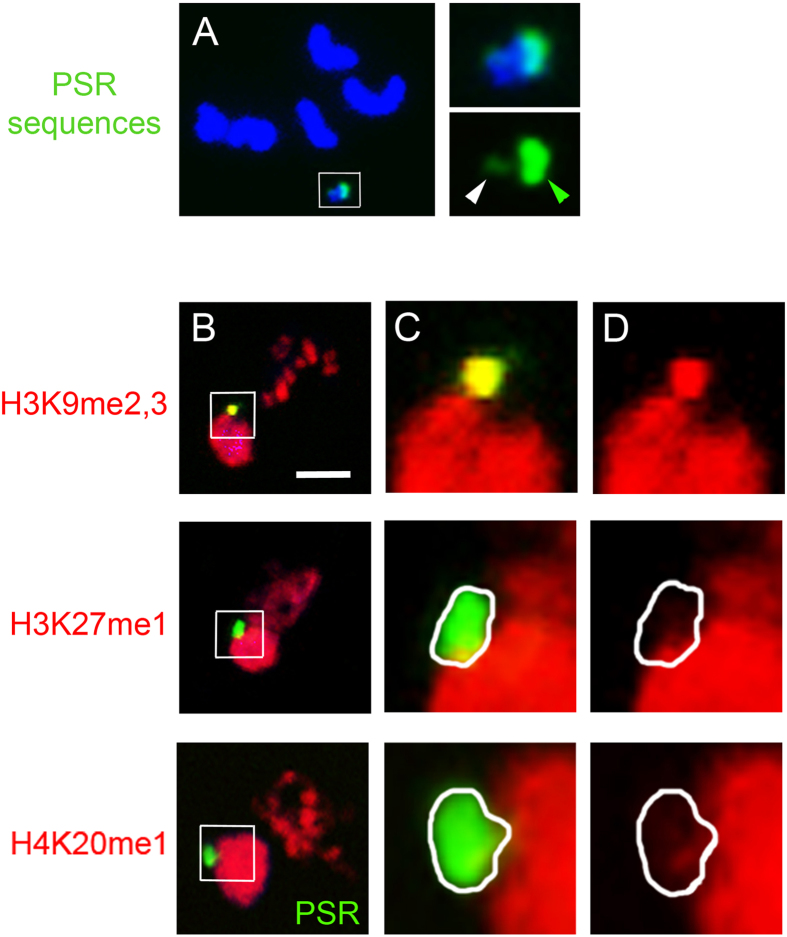
The PSR chromosome is devoid of H3K27me1 but not H3K9me2,3 and H4K20me1. (**A**) chromosome spread showing the five normal chromosomes in the *N. vitripennis* genome (top of panel) and the PSR chromosome (bottom of panel, in white box). Right panels show a higher magnification of the PSR chromosome from left panel, hybridized with a DNA FISH probe that highlights a satellite sequence that is present across most of the larger arm (green arrowhead) and in a small region on the shorter arm (white arrowhead). (**B**,**C**,**D**) PSR stained for the three histone marks H3K9me2,3, H3K27me1, and H4K20me1, respectively. Middle and far right column are higher magnifications of PSR, with the middle panels depicting the histone mark and PSR FISH probe. The far right panels show the same images without the PSR FISH probe in order to clearly visualize whether PSR contains a given histone mark. PSR is circumscribed (white line) in middle and bottom rows for clarity. Scale bar equals 12 μM.

Any of the three perturbed histone marks on the PCM, or a combination of them, could play a role in blockage of chromosome resolution and paternal genome loss. Because PSR is excluded from this effect, we speculated that PSR would likely avoid any such histone mark alteration(s). To test this possibility, we closely scrutinized the PSR chromosome during metaphase, the time when all three perturbed histone marks are clearly visible on the PCM. In particular, we were able to clearly highlight the PSR chromosome by hybridization with a probe that recognizes repeat sequences that span most of PSR’s long arm and a small region on its short arm (Fig. 6). H3K9me2,3 appeared to be at levels on PSR that are higher than those present on the PCM but comparable to levels present on the pericentromeric regions of the maternal chromosomes (Fig. 6). However, strikingly, very low levels of H3K27me1 and H4K20me1 were present on PSR during the first mitotic division (Fig. 6). Thus, PSR has the unusual ability to largely exclude itself from these two marks. This finding implicates H3K27me1 and H4K20me1 as important, perhaps in conjunction with H3K9me2,3, for subsequent H3S10p and Condensin misbehavior. Additionally, largely reduced levels of H3K27me1 and H4K20me1 on PSR likely help it to resolve and segregate properly during the first mitosis, thereby avoiding self-elimination.

## Discussion

An important aspect of understanding PSR-induced genome elimination is to discern how PSR blocks transformation of the paternal chromatin into chromosomes during the first mitotic division of the embryo. In this study we investigated whether PSR induces this effect directly or instead secondarily by disrupting earlier aspects of chromatin remodeling that occur immediately after fertilization. Our results support the later scenario. Specifically, we found that three post-translational histone modifications, H3K9me2,3, H3K27me1, and H4K20me1, become visible at their normal times on the paternal chromatin, but they spread ectopically across the entire paternal genome where they appear unusually intense – a pattern that persists through the first mitosis. This effect appears to be somewhat specific because a number of other histone marks, including H4ac, H3K4ac, and H3K27me2,3, were not visibly perturbed by PSR. Additionally, these histone mark perturbations occur before the previously reported abnormal behavior of H3S10p during the first mitotic division^7^. Thus, it appears that failure of the paternal chromatin to form individual chromosomes stems from earlier defects in the chromatin state.

How might perturbation of these histone modifications translate into blockage of chromosome formation? At the outset of this study we entertained the possibility that PSR induces heterochromatinization of the paternal set. This idea was initially supported by our observation that H3K9me2,3, a key mark of constitutive heterochromatin, spreads abnormally across the paternal set. It is well known from other studies that HP1 associates with H3K9me2 and H3K9me3 and also interacts with SU(VAR)3-9, an enzyme that di- and tri-methylates H3K9. These interactions are thought to establish a positive feedback loop that maintains constitutive heterochromatin^36^,^37^. However, by visualizing the single HP1 protein present in the *N. vitripennis* genome, we found no visible HP1 on the PCM or the maternally derived nuclei, although this protein localizes brightly to chromocenter-like regions of the nucleus during later embryonic stages. These findings argue that PCM formation does not involve heterochromatinization.

Our studies suggest that no single PSR-altered chromatin mark leads to paternal genome elimination. Indeed, the PSR chromosome, which successfully segregates during the first mitotic division despite being a component of the paternally derived nucleus until the initial anaphase, harbors one of three histone marks that are ectopically placed on the PCM before the first mitosis. This finding supports the idea that a combination of three or more altered histone marks leads to paternal genome elimination. This idea is consistent with evidence suggesting that chromatin states are determined not by individual marks but instead by combinations of certain ones. For example, transcriptionally active regions can simultaneously harbor acetylated histone H3K9, mono-methylated H3K27 and mono-methylated H3K4, in addition to other marks^21^,^38^,^39^. Such histone mark combinations may constitute a ‘code’ that provides complex input for association of certain combinations of chromatin-associated structural proteins and chromatin-modifying enzymes^20^,^21^. To our knowledge, the combination of histone marks that are disrupted by PSR in *N. vitripennis* is unique, not matching any previously characterized chromatin sub-type. While H3K9me2 and H3K9me3 are signatures of constitutive heterochromatin, H3K27me1, while not as well studied, is thought to be a transitional state toward H3K27me3, a mark of facultative (*i.e*., non-permanent) heterochromatin. Incidentally, our experiments have shown that the paternally inherited genome is visibly devoid of H3K27me2,3 in both the presence and absence of PSR. The H4K20me1 mark has been implicated in chromatin compaction; mutational loss of the enzyme that produces this mark, PR-Set7, results in abnormal nuclear and chromosome morphology. Based on this information, we propose that the altered combination of H3K9me2,3, H3K27me1, and H4K20me1, and perhaps others not uncovered in this study constitute a ‘misspelled word’ in the histone code, thereby causing an altered chromatin state of the paternal set that is not conducive to chromosome resolution. Support for this idea stems from the fact that experimental manipulation of these marks individually by mutation of the enzymes that produce them results in chromatin defects and genome instability^40^,^41^. Our finding that replication-licensing factors fail to localize to the paternal chromatin following the appearance of the defective histone marks in the presence of PSR further argues that the chromatin state of the PCM is substantially altered.

We further speculate that because PSR itself shows very low levels of two of these altered histone marks, its resolution and segregation are not similarly affected. PSR’s lack of H327me1 and H4K20me1 could be explained in part by its unique sequence composition. Previous cytological mapping has shown that PSR carries a unique set of non-coding sequences, including multiple complex satellite repeats, and at least one transposable element known as NATE^42^,^43^,^44^. Studies of PSR derivatives deleted for certain chromosomal regions suggest that the affected satellite repeats or other sequences in these regions may contribute to the genome elimination properties of PSR, or its ability to segregate properly, or both^45^. However, these sequences may also possess a unique chromatin state that is recalcitrant to the H3K27me1 and H4K20me1 marks. Another aspect to consider is that because PSR is a centromeric fragment, it lacks euchromatic ‘arms’ that are present on the five normal chromosomes in the wild type *N. vitripennis* genome. Thus, it is possible that alteration of the histone code specifically within the wasp’s euchromatin initiates the previously observed chromatin defects and helps PSR to evade this fate. Another possible explanation for PSR’s avoidance of self-elimination is that the B chromosome sequesters itself within a sub-nuclear domain that excludes certain chromatin-modifying enzymes. Support for this latter idea stems from previous studies showing that PSR obtains a distinct position within the elongated nucleus of mature sperm, and also resides at the outer periphery of the paternal nucleus following fertilization^7^. In addition, extrachromosomal entities, such as micronuclei formed from genotoxic stress conditions, are known to be surrounded by lamin-containing membrane^46^. Such a mechanism may aid PSR in obtaining some level of physical separation from certain chromatin modifying enzymes. However, such an idea should be further investigated by examination of the nuclear envelope with fluorescence and ultrastructural studies.

The results presented here strongly suggest that the initial-most alteration of the paternally-derived chromatin occurs during the period of embryogenesis immediately following the first S-phase, when the paternal chromatin becomes repackaged with histones and certain histone marks are placed in order to establish different chromatin environments. Interestingly, all three altered histone marks uncovered in this study play a role in normal remodeling of the paternal chromatin in wild type embryos. In contrast, one of the examined histone marks, H3K27me2,3, which is not present on the paternal chromatin in the wild type genotype, was not ectopically induced on the paternal chromatin by PSR. Thus, it appears that PSR may only alter chromatin pathways that are normally a part of paternal genome remodeling during early embryogenesis. Disruption of these pathways is an effective way of affecting the paternal chromatin to the exclusion of the maternal set, primarily because the maternal chromatin does not undergo protamine-to-histone repackaging and *de novo* establishment of histone marks. Direct targeting of paternal chromosome resolution would, in principle, be a more problematic scenario given that both paternal and maternal sets undergo this process.

How might PSR physically disrupt the placement of histone marks on the paternal chromatin? A distinct enzyme establishes each of the affected histone marks. For example, as stated above, mono-methylation of H4K20 is induced by PR-Set7^47^,^48^. Likewise, di- and tri-methylation of H3K9 stem from the activity of Su(Var)3-9^49^, and methylation of H3K27 is placed by the Enhancer of Zeste or E(Z), a member of the Polycomb family^50^,^51^. Our work demonstrates that the histone marks placed by these enzymes occur in ectopic regions spanning the entire paternal chromatin, in contrast to their being more restricted to sub-regions as is the case in the wild type state, and they appear to be more intense than normal. We speculate that PSR produces one or more products that direct the enzymes to these ectopic regions where their activities may not be properly regulated. Interestingly, a previous study identified nine polyadenylated transcripts with poor coding potential that are produced from PSR in the testis^43^. While much is still to be learned regarding the potential function(s) of these RNAs, it is possible that one or more of them may be transmitted to the embryo within the sperm nucleus, where they could facilitate the redirecting of histone modifying enzymes to improper regions of the paternal chromatin. This idea is supported by previous work in mice and maize demonstrating that non-coding RNAs transported by sperm (or pollen) can induce paramutation, an epigenetically inherited state of allele silencing, in progeny through multiple generations^52^,^53^,^54^. Additionally, it has been proposed that paternally transmitted small RNAs may provide a means for establishing *de novo* histone mark patterns in the paternal chromatin as it undergoes repackaging in a sequence-specific manner by acting as guides for histone- and DNA-modifying enzymes^55^,^56^. Currently it is not known if PSR produces its own unique subset of small non-coding RNAs. However, such small RNAs, or long RNAs produced by PSR, may play a similar role by misdirecting histone enzymes involved in paternal chromatin remodeling to ectopic sequences in the paternal genome. Such a mechanism may affect multiple different histone-modifying enzymes simultaneously, or instead, a single enzyme may initially be misdirected, thereby leading to subsequent, secondary misdirection of other enzymes. The nature of our cytological data does not allow us to distinguish between these possibilities.

The strikingly different sub-nuclear behaviors of PSR and the paternal genome, while fascinating, collectively represent a general scenario that is in one sense not entirely novel. For example, in female mammalian embryonic cells, one of two X chromosomes undergoes chromatin reprogramming through the actions of a long non-coding RNA named Xist and other non-coding RNAs in order to undergo transcriptional silencing^57^. This effect is, in large part, restricted to a subdomain within the nucleus that is defined by the inactivated X chromosome and therefore does not affect the active X chromosome or the autosomes (*i.e*., non-sex chromosomes). Thus, X inactivation, like PSR-induced genome elimination, stands as a clear example of extreme positional difference in chromatin dynamics within the same nucleus. However, PSR’s behavior is different from X inactivation in two basic ways. First, whereas X inactivation is an essential cellular event that allows for proper dosage compensation of X-linked genes in females, PSR is a non-essential part of the genome, which not only does not contribute to the fitness of *N. vitripennis* but, moreover, is deleterious to the wasp, completely destroying the paternal half of its genome and thereby drastically altering the normal sex ratio within populations of this organism^58^. Second, X inactivation occurs *in cis* through the function of Xist, which is expressed from a locus that is present on the X chromosome^57^. Although the molecular mechanism of PSR-induced genome elimination is currently unknown, PSR acts *in trans* on the paternal genome.

To summarize, we found that the ‘selfish’ PSR chromosome specifically alters the patterns of three histone marks that are aspects of paternal genome remodeling after fertilization in the jewel wasp. Other histone marks and DNA methylation are not disrupted by PSR. Thus, PSR disrupts specific aspects of the wasp’s epigenetic programming at this very early developmental time in order to induce genome elimination. These findings provide discrete cellular factors, including histone modifying enzymes, as potential targets that can be experimentally tested. Identification of the specific molecular targets, and the molecular means by which PSR alters these targets, will be important for understanding how PSR-induced genome elimination and other forms of intragenomic conflict can arise from a functionally unified genome.

## Methods

### Wasp lines and crosses

Crosses for PSR-carrying lines were conducted by crossing wild type virgin AsymC females and males of the same genetic background that carried the PSR chromosome. Virgin females were collected as pupae and isolated from their male siblings until eclosion. Equal numbers of females and males (approximately 20 each) were placed together in glass vials and allowed to mate for 1–2 days at 25 °C in a humidified incubator and fed with 50% honey in water and 2–3 *Sarcophaga bullata* blowfly pupae. For propagation of PSR-carrying lines, a subset of these mated females (10–15) were placed individually into glass vials and given 2–3 hosts, and allowed to lay embryos for 3 days and then removed. Propagation of the wild type line was conducted by setting eclosed sibling male and female wasps in equal proportions onto blowfly pupae.

### Embryo collection and fixation

For embryo collection, blowfly hosts were removed from mated wasps the day before embryo collection in order to prevent females from extinguishing their embryo supplies prematurely. Groups of 3–4 mated females were placed into individual glass vials and allowed to oviposit into a single blowfly pupa for discrete lengths of time. Specifically, a 45 min-1 hr laying time was used for obtaining embryos staged between fertilization and immediately before the first mitotic division, whereas a laying time of 1 hr 15 min was used for collecting embryos undergoing the first mitotic division or in the second S-phase.

Embryos were carefully removed from host pupae by using one half of a separated pair of ultrafine forceps and placed into a 10 mL screw top glass vial. The following solutions were quickly placed into the vial with embryos in the following order: 3 mL heptane; 1.5 mL 1x Phosphate Buffered Saline (1x PBS); 600 μL 37% formaldehyde. The vial with embryos in fixative was placed onto a platform rocker and embryos were fixed for exactly 28 minutes. Following this time, embryos were removed and placed onto a small piece of Whatman paper and allowed to dry for ~30 sec to 1 min. The embryos were lightly pressed downward onto double-sided adhesive tape secured to the surface of a clean 22 mm Petri plate. 1.5 mL of 1x PBT (1x PBS with 0.1%Triton-X 100) was then placed into the Petri plate to keep the embryos hydrated. The embryos were carefully devitellinized under a dissecting microscope by using a 28-gauge hypodermic needle. The devitellinized embryos were transferred to a 0.6 mL microfuge tube, washed three times with 1x PBT and stored at 4–6 °C before staining.

### Immunostaining

Primary antibodies used in this study and their dilutions in 1x PBT were: 1:500 for rabbit anti-panH4ac (Millipore), rabbit anti-H3K9me2,3 (Active Motif), rabbit anti-H3K9me2 (Active Motif), rabbit anti-H3K9me3 (Active Motif), rabbit anti-H3K27me1 (Active Motif), rabbit anti-H3K27me2,3 (Active Motif), rabbit anti-H4K20me1 (Active Motif), rabbit anti-H3K4ac (Active Motif); 1:250 for mouse anti-5′-methylCytosine (Abcam); 1:500 for rabbit anti-ORC2 and 1/100 for mouse anti-MCM2-7 (gifts from D. and H. MacAlpine).

To detect HP1 we produced a polyclonal rabbit serum against three KLH-conjugated peptides (KDSPSTEGETEE; KSSSTPTPTQSK; DDEGGHKPDPE) present in the single, full-length HP1 protein of *N. vitripennis*. Peptides were synthesized by SynPeptide CO, LTD (China). The serum was tested by Western blot analysis (see below) and immunofluorescence confocal microscopy (see Results). Embryo staining was conducted at an anti-HP1 serum dilution of 1:500.

Fixed embryos were stained with primary antibodies overnight on a platform rocker at 4 °C, washed 3 times at 10 minutes each with 1x PBT, and then stained with fluorescently conjugated secondary antibodies for 1 hr on a platform rocker in the dark. Secondary antibodies used in this study were: anti-rabbit Cy3 and anti-mouse Cy5 (both at 1:300; Invitrogen-ThermoFisher, Inc., USA). Embryos were then washed as above and either mounted on a slide with Vectashield mounting medium containing DAPI (Vector Laboratories, Inc., USA) or taken through the DNA FISH procedure (see below) before mounting. This general procedure was slightly modified for anti-5′-methylCytosine staining with the addition of a DNA denaturation step immediately prior to primary antibody staining. Embryos were treated with 50% formamide (diluted with 1x PBS) for 30 min in an 80 °C water bath before being washed 3 times with cold 1x PBT.

### Nuclear protein preparation and Western blotting

*Nasonia vitripennis* nuclei were prepared by lysing 250 uL of whole carcasses in 1 mL of solution A (0.25 M sucrose, 60 mM KCl, 10 mM NaCl, 10 mM 2-(N-morpholino) ethanesulphonic acid (MES) pH 6.5, 5 mM MgCl2, 0.5% Triton X-100 (w/v), 1 mM CaCl2), and sonicated 5 times with 10 sec on/off at high amplitude by using an Ultrasonic cell disruptor (Farmingdale, N.Y.), filtered through cheesecloth, centrifuged at 8700 × g for 5 minutes, and then resuspended in solution B (50 mM NaCl, 10 mM piperazine-N,N′-bis (2-ethanesulphonic acid) (PIPES), 5 mM MgCl2, 1 mM CaCl2) at a DNA concentration of 1.0 ug/uL. All the buffers contained Complete Protease Cocktail Inhibitors (Cat no: 11836145001, Roche Molecular Biochemicals, Laval, Quebec, Canada) at a dilution of 1:100. The final resuspended nuclei in solution B were dissolved in 2X SDS-PAGE buffer to a DNA conc. of 0.5 mg/ml, boiled at 95 °C for 3 minutes and loaded onto a 15% SDS gel.

Nuclear extracts were run on a 15% SDS poly-acrylamide gel at 100 V. A nitrocellulose membrane (0.2 μm, cat no. 162-0112 (BIO-RAD) was immersed in 1X Transfer Buffer [25 mM Tris base, 192 mM glycine, 20% Methanol]. Samples on 15% SDS gel were then transferred to the nitrocellulose membrane in 1X Transfer Buffer at 400 mA for 2 hours. Membrane was blocked with 3% (w/v) milk buffer PBS/0.1% Tween 20) for 1 hour at room temperature.

The primary rabbit polyclonal anti-HP1 antibodies were used in blotting at a dilution of 1:1000 and incubated overnight with agitation at 4 °C. For secondary detection, we used an ECL HRP-conjugated antibody (cat no. NA934, Mississauga, Ontario, Canada) at a dilution of 1:5000. Incubation of membrane with secondary antibodies was performed at room temperature for 1 hour and visualized using an Odyssey Licor Digit imager (LI-COR Biosciences, Lincoln, Nebraska).

### DNA *in situ* hybridization (FISH)

A small DNA probe used to detect PSR in fixed embryos was based on the following sequence that is exclusive to the PSR chromosome: 5′–CAC TGA AAA CCA GAG CAG CAG TTG AGA–3′. This sequence was chemically synthesized by IDT Inc. (USA) and fluorescently labeled at the 5′ end with Alexa-488. Before DNA FISH, immuno-stained embryos were post-fixed in the dark for 45 min in 4% paraformaldehyde and subsequently washed 3 times in 2x saline-sodium citrate and Tween-20 (SSCT). From this point, whole mount DNA FISH was conducted exactly as previously described^59^. For DNA FISH of squashed chromosomes, we followed exactly a previously described protocol, using testis as a source of mitotic chromosomes^60^.

### Confocal microscopy and image processing

Fluorescence microscopic imaging was conducted with a Leica TCS SPE confocal microscope. Images were collected as Z-series for each laser channel and subsequently merged for visual capture of cellular features within the same nucleus that were not in the same focal plane. Merged images were exported in highest quality JPEG format, and then remerged and processed in Adobe Photoshop CS5 v. 12.

## Additional Information

**How to cite this article**: Aldrich, J. C. *et al*. A ‘selfish’ B chromosome induces genome elimination by disrupting the histone code in the jewel wasp *Nasonia vitripennis. Sci. Rep.* **7**, 42551; doi: 10.1038/srep42551 (2017).

**Publisher's note:** Springer Nature remains neutral with regard to jurisdictional claims in published maps and institutional affiliations.

## Supplementary Material

Supplementary Information
