# Supplementary material for: A ‘selfish’ B chromosome induces genome elimination by disrupting the histone code in the jewel wasp Nasonia vitripennis
Source: Sci Rep. 2017 Feb 13;7:42551. doi: 10.1038/srep42551 (PMC5304203; doi:10.1038/srep42551)
Supplement: Supplementary Information [file srep42551-s1.pdf]

Title

A 'selfish' B chromosome induces genome elimination by disrupting the histone code in the jewel wasp Nasonia vitripennis

Authors

John Aldrich[1,&], Alexandra Leibholz[1,&], Manjinder S. Cheema[2], Juan Ausio[2], Patrick M. Ferree[1,*]

Supplemental Figure Legends

Figure S1. PSR-carrying embryos stained for individual histone marks H3K9me2, H3K9me3, H3K27me3, and H3K4ac. The PCM shows high levels of H3K9me2,3 and abnormally across its entirety (top panels). H3K27me3 (bottom left panel) marks the euchromatic arms of the normal chromosomes but not at all the PCM. Trace amounts of H3K4ac are present on both the maternally derived chromosomes and the PCM (bottom right panel). (p) and (m) mark the paternal and maternal sets, respectively. Scale bar equals 8 μM.

Figure S2. PSR disrupts H3K27me1 patterns on the paternal chromatin mass. In wild type embryos, H3K27me1 appears on the paternal set during nuclear juxtaposition but is already present on the maternal set (top, left-most panels). This mark persists in a similar intensity on both sets during the first mitosis and into the second interphase. In

PSR embryos, H3K27me1 appears during nuclear juxtaposition similarly to wild type embryos. However, this mark becomes extremely bright on the PCM and persists in this manner through the first mitotic division and into the second interphase. White arrowhead indicates the PCM. (p) and (m) mark the paternal and maternal sets, respectively. Scale bar equals 10 μM.

Figure S3. PSR disrupts H4K20me1 patterns on the paternal chromatin mass. In wild type embryos the H4K20me1 mark appears on the sperm's chromatin before juxtaposition (left-most column) and becomes slightly enriched on the paternal set relative to the maternal set during metaphase of the first mitotic division. However, by the second interphase, H4K20me1 levels are light and evenly distributed on both daughter nuclei. In PSR-carrying embryos, H4K20me1 appears normally on the elongated sperm nucleus but becomes abnormally bright and widespread across the paternal chromatin (white arrowheads) during metaphase of the first mitosis and into the second interphase. Scale bar equals 12 μM.

Supplemental Figures

Figure S1

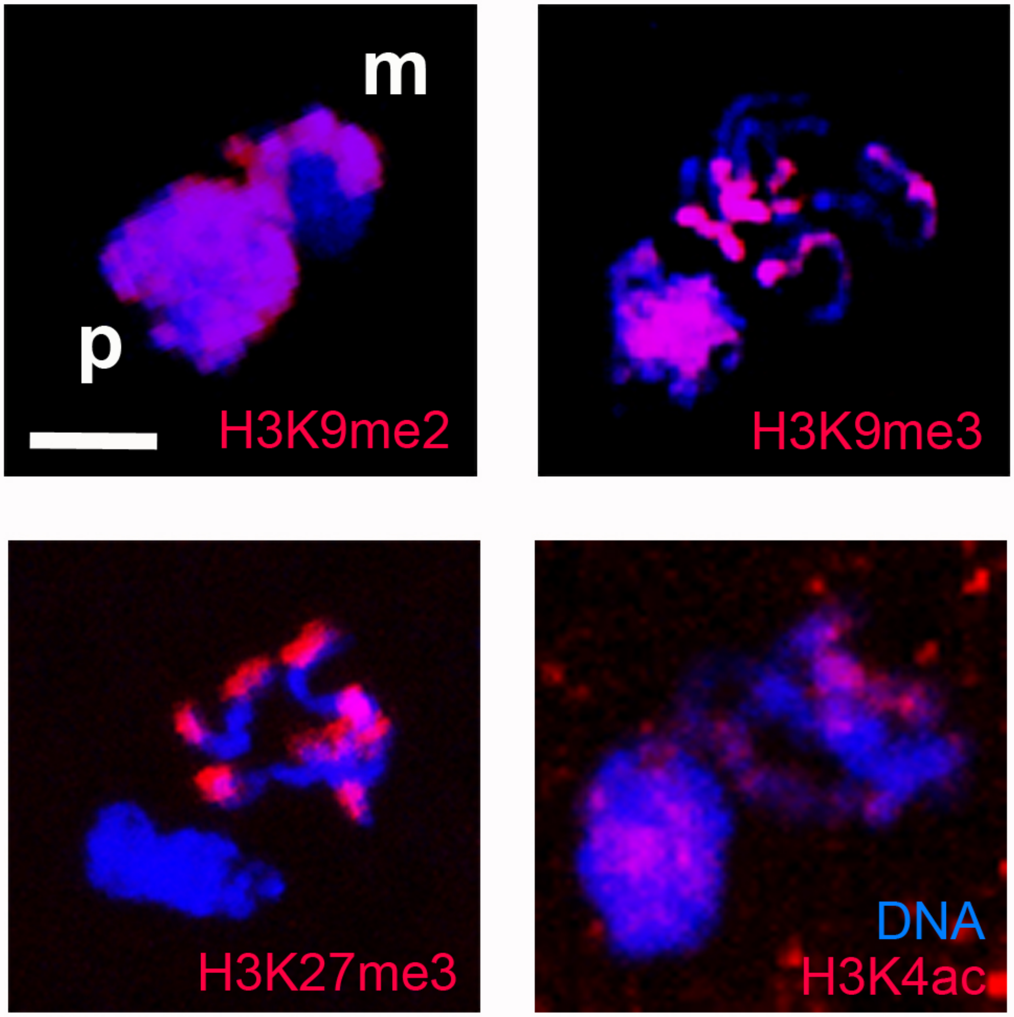

Figure S2

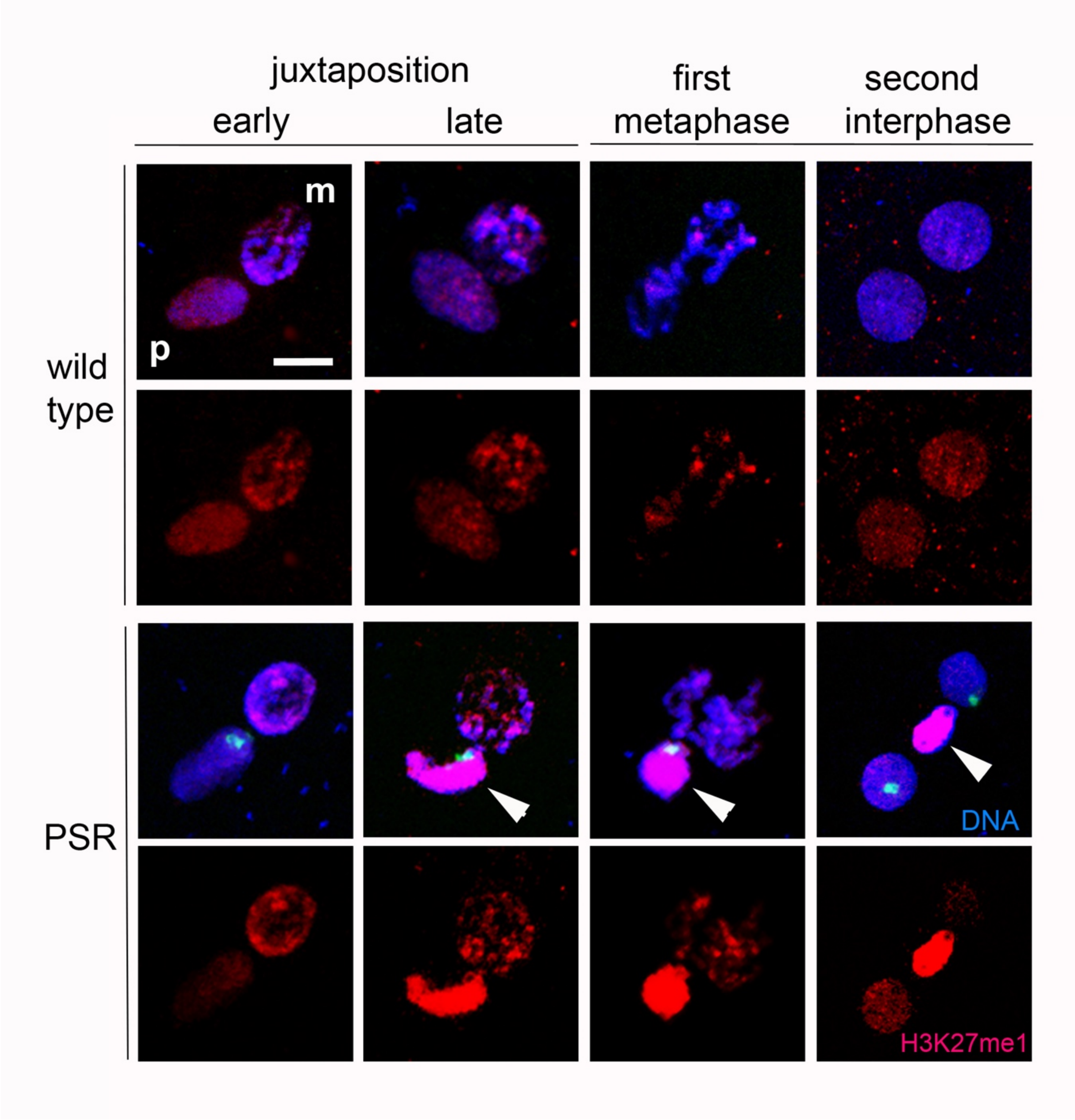
juxtaposition
early
late
first
metaphase
second
interphase
m
p
wild
type
PSR
DNA
H3K27me1

Figure S3

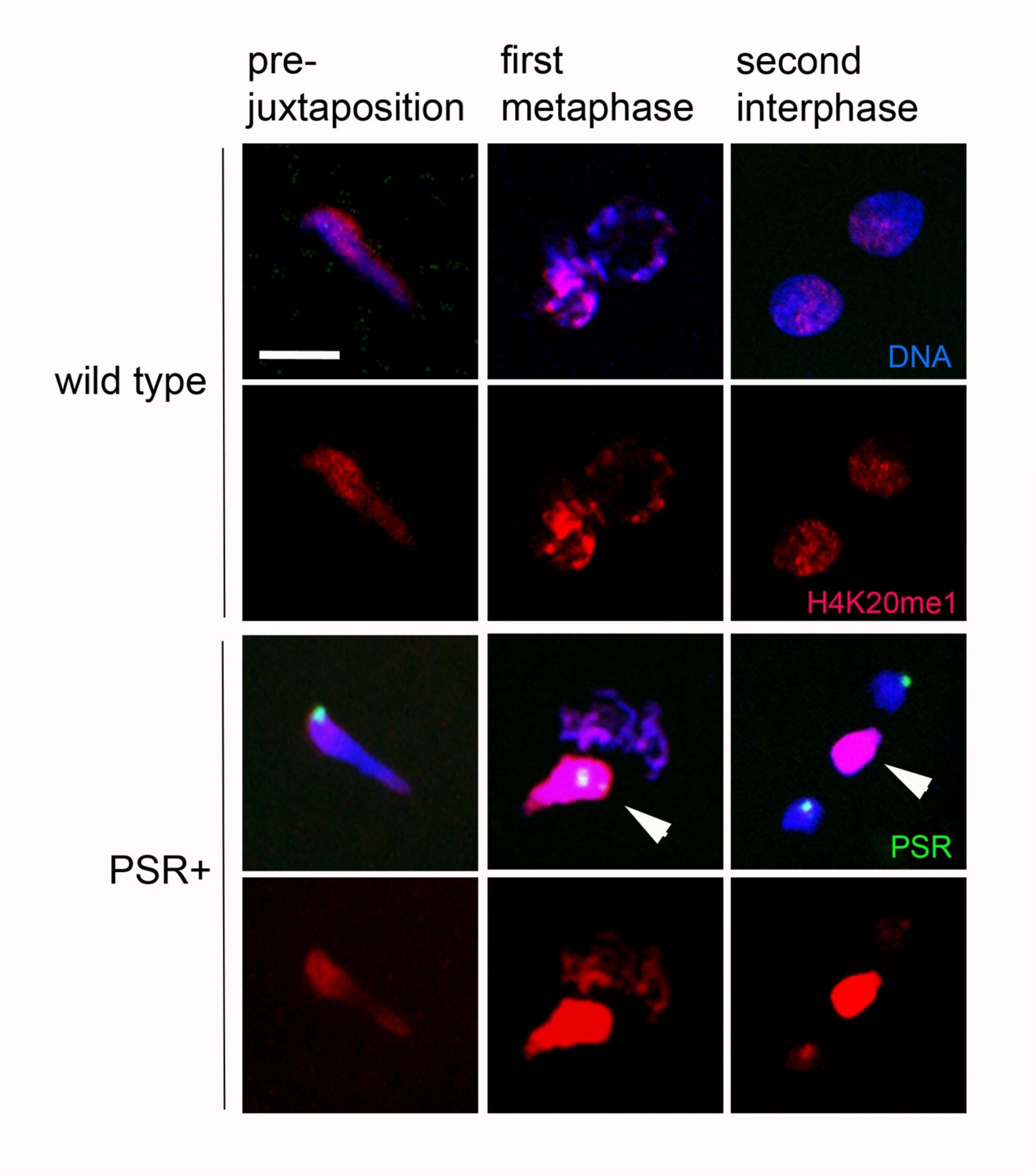
pre-
juxtaposition
first
metaphase
second
interphase
wild type
DNA
H4K20me1
PSR+
PSR
